# Controlling the Master: Chromatin Dynamics at the *MYC* Promoter Integrate Developmental Signaling

**DOI:** 10.3390/genes8040118

**Published:** 2017-04-11

**Authors:** Olga Zaytseva, Leonie M. Quinn

**Affiliations:** 1 ACRF Department of Cancer Biology and Therapeutics, The John Curtin School of Medical Research, The Australian National University, Canberra, ACT 2600, Australia; leonie.quinn@anu.edu.au; 2 School of Biomedical Sciences, University of Melbourne, Parkville 3010, Australia

**Keywords:** MYC, *Drosophila* dMYC, FUBP1/Psi, FIR/Hfp, TFIIH, Mediator, transcription, signaling, development, DNA topology

## Abstract

The transcription factor and cell growth regulator MYC is potently oncogenic and estimated to contribute to most cancers. Decades of attempts to therapeutically target MYC directly have not resulted in feasible clinical applications, and efforts have moved toward indirectly targeting MYC expression, function and/or activity to treat MYC-driven cancer. A multitude of developmental and growth signaling pathways converge on the *MYC* promoter to modulate transcription through their downstream effectors. Critically, even small increases in MYC abundance (<2 fold) are sufficient to drive overproliferation; however, the details of how oncogenic/growth signaling networks regulate *MYC* at the level of transcription remain nebulous even during normal development. It is therefore essential to first decipher mechanisms of growth signal-stimulated *MYC* transcription using in vivo models, with intact signaling environments, to determine exactly how these networks are dysregulated in human cancer. This in turn will provide new modalities and approaches to treat MYC-driven malignancy. *Drosophila* genetic studies have shed much light on how complex networks signal to transcription factors and enhancers to orchestrate *Drosophila MYC* (*dMYC*) transcription, and thus growth and patterning of complex multicellular tissue and organs. This review will discuss the many pathways implicated in patterning *MYC* transcription during development and the molecular events at the *MYC* promoter that link signaling to expression. Attention will also be drawn to parallels between mammalian and fly regulation of *MYC* at the level of transcription.

## 1. MYC: A Potent Driver of Cell and Tissue Growth

MYC upregulation is a key driver of tumorigenesis [1,2,3]. Fundamental to MYC’s impact on both normal development and cancer cell physiology is a potent capacity to promote cell and tissue growth through activation of the genes necessary for protein synthesis for accumulation of cellular biomass [4,5,6,7] and cell cycle machinery to drive proliferation [8]. MYC abundance therefore correlates with proliferation [9], consistent with the observation that MYC’s transcriptional activity decreases in differentiated cells [10,11]. MYC’s capacity to promote growth and cell division is essential for tissue patterning during organ growth and, given that elevated MYC is inexorably linked to cancer initiation and progression, developmental signals must normally ensure tight control of *MYC* expression [12,13].

MYC heterodimerizes with its partner bHLH protein MAX in order to bind DNA and activate transcription [14,15]. MYC-driven transcriptional activation is, in part, regulated by a related bHLH protein MAD which forms heterodimers with MAX, therefore limiting the pool of available MAX protein [15]. Other binding partners possess capacity to modulate MYC activity; for example, binding to MIZ-1 (ZBTB17) leads to transcriptional downregulation of MYC targets, providing a mechanism for MYC’s capacity as a transcriptional repressor of certain targets [16,17]. Early studies conducted in *Drosophila*, prior to the advent of genome-wide deep sequencing, implicated dMYC in controlling expression of 10%–15% of all genes [18,19]. The prominent and conserved transcriptional signature (i.e., cell growth and proliferation [4,5,6,7]) was attributed to MYC’s capacity to selectively upregulate a common set of cellular targets [13,20], enriched for the presence of the consensus “E-box” enhancer sequence [21,22]. Nevertheless, genome-wide chromatin immunoprecipitation (ChIP)-sequencing studies, detecting MYC on all promoters, enhancers and intergenic regions with an open chromatin structure [23,24,25,26], appear to challenge MYC’s role as gene-specific transcriptional regulator. Naïve mouse lymphocytes carrying endogenous MYC tagged with green fluorescent protein (GFP) were stimulated to induce *Myc* expression, revealing a global positive correlation between level of gene expression, active chromatin marks and MYC binding across the genome [23]. This observation is supported by studies in human Burkitt’s lymphoma tumour cell models [24], and together these studies suggest that MYC behaves as a broad amplifier of transcription. 

So how does the transcriptional signature associated with increased MYC arise in both normal and tumour cells? At the broadest level, global amplification as a result of elevated MYC levels will increase the output of previously active transcriptional programs, potentiating the growth and proliferation programs already operational in developing organs, proliferating cell culture systems or tumours. The observation that MYC binding may not always result in productive transcriptional outcomes [27] could also enable fine tuning of the MYC signature in the event of global binding to active genes. However, a recent study revealed that MYC’s differing affinity for certain promoters likely potentiates MYC-specific expression profiles [28]. Specifically, genes containing high-affinity promoters are predicted to be fully occupied by physiological levels of MYC; thus, introducing additional MYC protein can only increase binding at low affinity targets, generating a signature by enhancing expression of these weakly-bound MYC-targets. Together these studies explain why MYC has such potent oncogenic capacity. Although *MYC* overexpression alone does not induce aggressive tumours, in the context of dysregulated growth signaling, the ability of MYC to amplify all active transcription could lead to tumourigenesis. For instance, elevation of MYC will not only enhance cellular overgrowth and tissue hyperplasia (by binding promoters that are active in proliferating tumour cells), but will also upregulate expression of weakly expressed cancer-promoting targets to accelerate tumour progression. The expanse of MYC’s transcriptional capacity is reviewed comprehensively elsewhere [29], but the message is clear regardless of nuances; moderate increases in MYC can broadly influence the cell’s transcriptome. Thus, to maintain normal cell growth patterning during animal development, expression of *MYC* must be tightly controlled.

## 2. MYC Functional Redundancy Depends on Transcriptional Patterning

In mammals, the *MYC* family benefits somewhat from the redundancy of three family members, *MYC, MYCN* and *MYCL* (reviewed elsewhere [12,30]). *Myc* haploinsufficiency results in viable mice, which are small compared with wildtype due to impaired proliferation [31]. Some MYC is essential for viability, as null mice display severe developmental defects and die before midgestation [31]. Interestingly, the lethality associated with *Myc* nulls is actually a result of placental insufficiency, since embryo-specific knockout (in the epiblast) in the context of wild type extraembryonic structures (trophectoderm and primitive endoderm), generates embryonic organs without gross developmental abnormalities [32]. The exception is the hematopoietic lineage, which is particularly sensitive to *Myc* depletion; thus, placental rescue pups are severely anaemic due to loss of hematopoietic stem cells (HSCs) [32]. Strikingly, gene-replacement of *Myc* with *Mycn* results in viability, demonstrating that MYCN can provide functional compensation when MYC is absent [33].

The sensitivity of certain lineages to *Myc* depletion, and inability of *Mycn* expressed from its endogenous locus to compensate in the placenta and hematopoietic lineage, likely reflects large differences in expression levels of the paralogs between tissues. *Myc* is expressed broadly in developing mice, with particularly high levels of mRNA in thymus, spleen, and liver, while *Mycn* is highly expressed in the brain and kidney [34]. The cerebellum is more sensitive to conditional deletion of *Mycn*, which impairs neural stem and progenitor cell proliferation, while deletion of *Myc* does not alter these lineages [35]. In the tumour context, MYC and MYCN-driven medulloblastomas exhibit distinct phenotypes due to the preferential ability of MYC to inhibit expression of certain genes via binding to the MIZ-1 transcriptional repressor [36]. 

In the hematopoietic lineage, *Mycn* and *Myc* are only coexpressed in immature HSCs, and *Mycn* transcripts decrease following HSC differentiation [37]. In accordance with functional redundancy in early development, the *Mycn* and *Myc* double knockout hematopoietic phenotype is significantly more severe than individual knockout of either gene [37]. Although deletion of *Myc* prevents HSCs from differentiating into progenitors due to inability to exit the niche [38], the remaining endogenous MYCN enables maintenance of proliferation and self-renewal capacity, indicating that MYCN can provide all MYC-family functions during HSC maintenance. However, in contrast to *Myc*, deletion of *Mycn* alone does not impair HSC number or steady-state hematopoiesis [37]. Therefore, MYCN is only required in very primitive HSCs, while other functions are provided by MYC throughout the hematopoietic lineage.

Thus, despite the ability of MYCN to functionally replace MYC during development [33], and the observation that *Myc* depletion can lead to increased *Mycn* abundance [39], MYC and MYCN do not play overlapping roles in some mammalian organs. How, then, does the insertion of *Mycn* into the endogenous *Myc* locus rescue the *Myc* null? Expression of the *Mycn* gene from the *Myc* locus reflects the endogenous *Myc* patterning, suggesting that transcriptional control of the *Myc* locus is critical for normal development. 

## 3. Patterning *MYC* Transcription during Animal Development

In *Drosophila*, the single orthologue (*dMYC*) has enabled MYC function to be discerned without concern for redundancy. The functional conservation of MYC between invertebrates and vertebrates is stunning. Not only can expression of human *MYC* rescue lethal *dMYC* mutations in flies [40], but expression of *dMYC* can provide competitive advantage to *Myc* null mouse fibroblasts [39] and cooperate with activated *Ras^V1^*^2^ alleles to rescue proliferation defects and induce oncogenic transformation in *Myc* null rat fibroblasts [41]. Thus, like mammalian MYC, *Drosophila* dMYC regulates cell growth and progression through the cell cycle [42,43,44,45]. Indeed, *Drosophila* was the first organism where cell growth impairment phenotypes (i.e., a reduced cell size) were associated with dMYC reduction and diminished organ and tissue growth [42]. Specifically, hypomorphic *dMYC* mutant flies are smaller than wildtype, with normal organ proportions, albeit composed of cells reduced in size [42]. In line with the impaired growth, dMYC targets the machinery required for increasing cellular biomass, particularly for building ribosomes [6,46,47,48]. Conversely, global *dMYC* overexpression increases ribosome biogenesis and tissue growth to result in overgrown flies [6,49].

The potent oncogenic capacity of MYC is the downside of its efficiency to normally drive growth and proliferation in developing organisms, and once again highlights the need for precise regulation of *MYC* levels. Even small changes in MYC levels are sufficient for cell and tissue overgrowth [50,51], in contrast to other proto-oncogenes such as *Ras* or *EGFR* where mutations constitutively activate the protein [52]. To enable normal development, *MYC* expression must not only be tightly restricted, but also maintain capacity to rapidly respond to developmental growth signals according to the cell’s requirements. Since transcriptional upregulation of *MYC* expression can trigger and propagate tumour pathogenesis, we focused the next part of this review on the mechanisms orchestrating expression of *MYC* under normal circumstances and reflect on their disarray in the context of cancer.

## 4. Developmental Signals Patterning *MYC* Expression

For MYC to drive the major cellular growth and proliferation events orchestrating development, abundance is predominantly regulated at the levels of transcription and mRNA/protein stability. To attain the level of MYC appropriate to cell fate, signaling inputs to *MYC* transcription must be integrated with those altering mRNA and protein abundance. mRNA stability and translation of both mammalian *MYC* and *Drosophila dMYC* is modulated by numerous microRNAs (miRNAs) [53,54,55,56,57,58,59,60]. Further to this, the generally short half-life of the MYC protein [61] can be increased as a consequence of stabilising phosphorylation directly by kinases in the Ras/ERK pathway [62,63,64,65] and/or PI3K/AKT pathway via inhibition of GSK3-β, which, when active, targets MYC and dMYC for ubiquitin-mediated proteasome degradation [66,67,68,69]. Indeed, point mutations in the *MYC* coding sequence in the context of cancer [70,71] tend to cluster at the T58 phosphorylation site within the MYC transactivation domain. This can stabilise MYC protein as a result of reduced degradation [72,73], although certain point mutations at T58 do not extend the half-life of the protein [74], but might impair the ability of MYC to interact with regulatory binding factors to alter transcriptional activity.

Ultimately, increased MYC mRNA/protein relies on production of mRNA via transcriptional changes in response to extracellular stimuli and developmental growth signals. The orchestration of cell growth and cell cycle for tissue and organ development of multicellular organisms requires *MYC* patterning; *MYC* must be appropriately expressed in a subset of tissues and repressed in others. Early human cell culture studies demonstrated that *MYC* expression is rapidly activated by growth factors in serum, and maintained in proliferating stem cells/progenitor cells, but becomes downregulated in response to differentiation signals [75,76,77]. An overview of the multitude of developmental signals converging on the *MYC* promoter to pattern cell and tissue growth is outlined briefly below, but is more comprehensively reviewed elsewhere [44].

Links between upstream pathways controlling *dMYC* expression have been particularly well studied in the *Drosophila* wing imaginal disc, where dMYC drives cell growth and G1 to S phase progression [42,78,79]. The Wingless (Wg, Wnt pathway in mammals) morphogen drives cell cycle exit and differentiation in the wing by repressing *dMYC* expression [80]. On the other hand, activating Wg in the adjacent hinge region of the wing can drive tissue overgrowth [81], highlighting the complex outcomes elicited by the same developmental signal. Similar nuances in the orthologous Wnt pathway are observed in mammals, where the Wnt proteins can behave as oncogenes or tumour suppressors depending on cellular context. For instance, while most studies from the mammalian intestine highlight the capacity of Wnt to activate β-catenin/TCF4 binding to the *MYC* promoter in association with tissue overgrowth and tumour predisposition [82,83,84,85], Wnt signals drive terminal differentiation in Paneth cells in the intestinal crypt [86], inducing *Myc* in the crypt and *CyclinD1* in the villi distinct cell populations [87].

*Drosophila* studies have enabled the dissection of interplay between Wg/Wnt and the Notch pathway, another major architect of development frequently linked with tumour progression [88,89]. Notch can inactivate *dMYC* indirectly via the Wg/Wnt pathway to control *dMYC* expression, and thus promote cell cycle arrest and differentiation in the fly wing [90,91,92,93,94]. On the other hand, stimulation of the Notch pathway via overexpression of cleaved Notch intracellular domain promotes binding of the Su(H) transcription factor to the *dMYC* gene, increasing *dMYC* expression and inducing wing overgrowth independently of Wg/Wnt [95]. In *Drosophila* muscle cell precursors, Notch activation also increases *dMYC* expression [96]. Notch further refines *dMYC* expression via induction of the E(spl)m8 transcriptional repressor, which negatively regulates *dMYC* in the wing [95]. In the context of gastric cancer, NOTCH4 induces Wnt signaling to result in activation of *Myc* by β-catenin [97]. Mammalian NOTCH1 can also directly activate *Myc* transcription by interacting with the Su(H) orthologue CBF1 in murine mammary cells [98]. Moreover, *MYC* is an essential NOTCH1 target for tumour progression in mammalian T cell lymphoblastic leukaemia (T-ALL) cells [99] and mouse models [100,101], where the *Myc* promoter is directly activated as a result of binding by intracellular domain of NOTCH and CBF1. In mouse models for pancreatic neoplasia, specific NOTCH2 activation of *Myc* is also required for tumour development [102].

Further to the responsiveness of the *MYC* promoter to the master developmental regulators WNT and NOTCH, a myriad of cellular signalling cascades possess capacity to influence *MYC* transcription. In the wing disc, the Decapentaplegic (Dpp, TGFβ in mammals) morphogen controls *dMYC* expression, albeit indirectly by downregulating the transcriptional repressor Brinker (Brk), which normally acts to suppress *dMYC* [103]. Testament to the context dependency of such morphogens, TGFβ can also repress mammalian *MYC* via direct activity of the downstream target SMAD3/SMAD4 on the *MYC* promoter [104]. In *Drosophila* midgut, JAK/Stat and EGFR activity increase *dMYC* promoter activity [105], while the mammalian *MYC* promoter is activated in response to JAK signaling, but independently of STAT3 [106]. The insulin/TOR pathway induces *MYC* expression via direct binding of the downstream transcription factor FOXO [107]. *Drosophila dMYC* transcription is also activated directly by the downstream effector of Hippo pathway, Yorkie (Yki) [108], and overexpression of the mammalian Yki orthologue, YAP, increases MYC abundance in mouse models [109]. The functional conservation and utility of *Drosophila* genetics has enabled dissection of complex networks controlling MYC abundance in multicellular organisms under conditions of normal development, thus enhancing our understanding of the mechanisms of MYC dysregulation in disease.

## 5. *MYC* Promoter Architecture Reflects Signaling Inputs

Early interest in *MYC* promoter architecture came from the observation that MYC-driven malignancy was often associated with rearrangements of the *MYC* locus and, thus, dysregulation of transcription. In particular, the B-cell malignancy Burkitt’s Lymphoma is associated with reciprocal chromosomal translocations between *MYC* and one of 3 immunoglobulin loci [110,111]. The breakpoints relative to both *MYC* and the immunoglobulin loci vary considerably, being either internal to the *MYC* transcriptional unit or up to several hundred kilobases away. Interestingly, the translocated *MYC* alleles contain either truncated or mutated exon 1, which alleviates the tightly regulated attenuation of RNA Polymerase II (Pol II) transcriptional elongation [112], enabling constitutive Pol II transcriptional read-through and increased *MYC* expression [77,113,114]. In accordance, a block to Pol II elongation in the endogenous *MYC* promoter induces transcriptional down-regulation of *MYC* and differentiation in human leukaemia cell lines [115]. This block to progression of engaged Pol II, or promoter proximal paused Pol II, was first interrogated using models of the human *MYC* promoter [77,116]. In vitro studies using an intact *MYC* locus identified Pol II accumulation downstream of the *MYC* transcription start site, engaged but unable to proceed to productive elongation [117]. As *MYC* receives multiple activation signals, post initiation control via Pol II pausing provides opportunity for rapid transcriptional activation in response to developmental signaling, which could not be achieved by Pol II recruitment alone.

## 6. FUBP1—A Hyperactivator of *MYC* Transcription

At promoters, melting of duplex DNA is obligatory for Pol II entry and transcriptional initiation. Indeed, DNase hypersensitivity of the human *MYC* promoter correlates with activity, being more sensitive in cells actively transcribing *MYC* compared with *MYC*-silent cells [77,115,118]. Thus, major changes in DNA architecture, particularly generation of nuclease-sensitive, non-B DNA elements, will arise as the multitude of growth and developmental signaling networks converge on the *MYC* promoter. The disruption to DNase hypersensitive elements in Burkitt’s lymphoma cells [115] led to the characterization of chromatin modifications and mapping of the DNA elements regulating the endogenous *MYC* promoter as a means of understanding mechanisms of this MYC-driven cancer. Of the many sequence-specific binding sites predicted for the *MYC* promoter based on the nuclease sensitivity assays, induction of differentiation and downregulation of *MYC* expression is only associated with lost binding activity of the far upstream sequence element (FUSE) 1.5kb upstream of the P1 promoter [119]. 

Oligonucleotide affinity chromatography using the FUSE retrieve the FUSE binding protein (initially FBP and recently renamed FUBP1) from proliferating cell extracts, with binding dramatically decreasing following induction of differentiation and down regulation of *MYC* [120]. Preferential FUBP1 binding to the noncoding, single-stranded FUSE provoked a closer examination of FUSE structure and function. Early studies revealed broad regions of specific S1 nuclease (single-strand nucleic acid) sensitivity in chromatin upstream of the human *MYC* gene [76]; however, mapping using potassium permanganate, which reacts preferentially with thymine in single-stranded DNA, enables single-base resolution [121]. Indeed, such permanganate assays were used in the first studies confirming melting of DNA strands in transcription bubbles associated with paused Pol II downstream of P2 promoter in human *MYC* [122]. Permanganate mapping of the *MYC* coding strand reveals hyperreactive thymidine residues in FUSE, consistent with an open single strand extending from FUSE toward P1 [121]. In contrast, the noncoding strand is predominantly hyporeactive; particularly protected are the nucleotides preferentially bound by FUBP1 in vitro, i.e., consistent with FUBP1 binding the single-stranded noncoding strand of FUSE in vivo [120]. Interestingly, FUBP1 is able to alter DNA conformation, possessing ability to force separation of the FUSE contained in supercoiled plasmid DNA and drive further opening of dsDNA at distances over 2.8 kilobases away [121]. Indeed, subsequent studies (described below) demonstrated that FUBP1-dependent remodeling of the *MYC* promoter structure is essential for tight regulation of *MYC* transcription.

The observation that FUBP1 binds the single-stranded FUSE, in preference to the double-stranded sequence, suggests that formation of the FUBP1-DNA complex requires prior unwinding of the DNA helix [123]. In accordance, FUSE is contained within a region of helical instability predicted to partially unwind in negatively supercoiled DNA, which would provide a platform for more extensive double-strand separation and stabilisation driven by FUBP1. Strand separation associated with targeted melting of the A-T rich FUSE, and subsequent binding activity of FUBP1, would further drive supercoiling and generate torsional energy within adjacent double-stranded DNA (dsDNA) strands. Indeed, double-strand opening is only observed in vitro when FUSE is surrounded by supercoiled—not unwound—double-stranded plasmid DNA [123]. As conversion of negatively supercoiled plasmid to the relaxed form using Topoisomerase I (Topo I) abolishes all spontaneous and FUBP1-induced strand melting, the helical stabilisation effect on FUSE in supercoiled DNA must be a consequence of non-B-DNA induced by FUBP1 at the more distant sites. Analysis of the interaction between FUBP1 and torsionally stressed supercoiled DNA, but not linear duplexes, suggests FUBP1 might directly link alterations in DNA conformation and topology with changes in *MYC* expression [121]. Factors that recognize topological strain, such as FUBP1, can thus act as sensors of *MYC* promoter activity. Under the circumstances where *MYC* transcription initiates (i.e., Pol II engages with the open P1 and P2 duplex but does not immediately escape the promoter) FUBP1 binding to this pre-activated *MYC* promoter would enable release of the paused Pol II to drive promoter escape and enhance transcriptional elongation. 

Hence, in addition to conventional dsDNA-binding transcriptional regulators, activity of single-strand nucleic acid binding proteins can drive *MYC* transcription. Moreover, energy generated from promoter unwinding can be harnessed as a productive force in *MYC* transcription [124]. Specifically, increased *MYC* expression will be associated with region-specific destabilisation of B-DNA in torsionally strained regions of the active *MYC* promoter, and forward movement of Pol II will generate and transmit negative supercoils to the FUSE to enable FUBP1 binding. Thus, *MYC* promoter activity associated with transcription initiation enables FUBP1 binding to FUSE to maximize Pol II release and activation of *MYC* transcription [125]. 

## 7. Developmental Function of FUBP1

Interaction between FUBP1 and the single-stranded FUSE is essential for maximal activation of the *MYC* promoter; however, the physiological outcomes of single-strand-specific FUBP1 binding during development is still relatively obscure. Early studies demonstrated that reduced FUBP1 levels and/or activity diminishes *MYC* expression and cell proliferation in ex vivo cell culture systems [126]. FUBP1 knockdown in human hepatocellular carcinoma (HCC) cell lines also decreases proliferation, and impairs tumour formation in mouse xenograft models [127]. Essential functions in HSC self-renewal were revealed using *Fubp1* gene trap mice, with embryonic lethality (around E15.5) associated with anaemia [128]. Secondary transplantation assays for long-term repopulating hematopoietic stem cells (LT-HSCs) revealed reduced blood cell reconstitution for *Fubp1* knockdown. *Fubp1*-deficient adult HSCs exhibited increased expression of key cell cycle (cyclin-dependent kinase inhibitor, *p21*) and pro-apoptotic (*Noxa*) genes based on mRNA expression profile data [128]. Given that the haematopoietic lineage displays specific sensitivity to MYC levels [32], and the ability of MYC to repress *p21* expression [129], the *Fubp1* knockout phenotype suggests that *MYC* could be a key downstream target that mediates these effects. Further studies are therefore required to determine if these transcriptional changes are direct, or occur indirectly, via altered *MYC* expression.

*Fubp1* knockouts display variable penetrance; although mice start dying at embryonic day 10.5 they can survive until birth [130]. Broader phenotypic analysis revealed a diverse range of defects including small body size, pulmonary hypoplasia, hypoplastic spleen/thymus/bone marrow, cerebral hyperplasia, pale livers, cardiac hypertrophy and placental distress, all characteristic of severe anaemia. The *Fubp1* knockout had normal numbers of HSCs, but transplantation of *Fubp1* loss-of-function HSCs into irradiated mice failed to reconstitute haematopoiesis [130]. The discrepancy with the *Fubp1* gene trap, which reduces but does not eliminate reconstitution capacity [128], suggests the gene trap might be hypomorphic. 

Interestingly, *Myc* mRNA levels varied dramatically between individual ex vivo cultures of *Fubp1* knockout mouse embryo fibroblasts (MEFs) harvested from different *Fubp1* embryos [130]. The reason for this variability is currently unknown, but it is possible that reduced FUBP1 binding on *Myc* impairs recruitment of FIR (FUBP-interacting repressor, discussed below) and results in a failure to shut *Myc* down, thus generating transcriptional noise. Another possibility is redundancy and/or compensatory activity of the other FUBP family members, FUBP2 and FUBP3. Like FUBP1, FUBP2 and FUBP3 can bind sequence specifically to the non-coding strand of FUSE, and possess potent transcriptional activation domains in vitro [131]. FUBP2 would be predicted to be most likely to compensate for loss of FUBP1 function as these two family members share the most functional similarity; in contrast, FUBP3 is only weakly localised to the nucleus and does not bind FIR [132]. Given FUBP1 and 2 regulate common target genes in vivo, including *MYC,* it will be of great interest to determine whether FUBP1 loss-of-function leads to heightened FUBP2 activity and the phenotypic outcome of double knockout. In short, these mouse studies leave the major function of FUBP1 during development requiring clarification. 

## 8. FUBP-Interacting Repressor (FIR)—the FUBP1 Antagonist Keeps *MYC* Quiet

Yeast two-hybrid screens, using FUBP1 as bait, identified the FUBP1 interacting repressor (FIR) as a potent binding partner [133]. FIR behaves as an FUBP1 antagonist, blocking activator-dependent, but not basal *MYC* transcription. A co-immunoprecipitation (co-IP) screen in HeLa cells using purified components of the basal transcription apparatus available at the time (i.e., the general transcription factors or GTFs) detected association of FIR with TFIIH, but not with GTF complexes necessary at earlier stages of promoter recognition and preinitiation complex (PIC) formation [133] (e.g., TBP, TAFs, TFIIB, TFIIF [134,135,136]). However, FIR did not alter TFIIH-mediated phosphorylation of the carboxyl-terminal domain (CTD) of the largest Pol II subunit at S5 [133] required for initiation and promoter escape [137,138,139], so was unlikely to cause repression of the cyclin-dependent kinase 7 (CDK7) kinase module of TFIIH. Rather, FIR inhibited the helicase activity of TFIIH’s xeroderma pigmentosum type B (XPB)/P89 module, which is essential for both transcription initiation and promoter escape [140,141,142].

FIR therefore blocks FUBP1-dependent *MYC* activation by decreasing XPB helicase activity, to permit only basal transcription [133]. The *MYC* promoter integrates multiple signaling inputs, but regulation by TFIIH at the level of promoter clearance would provide an opportunity to control transcription post-initiation. Repression of TFIIH by FIR, late in the transcription cycle, would provide an additional regulatory mechanism to safeguard against inappropriate or excessive signal-induced *MYC* activation. In context with the post-initiation block controlling *MYC* transcription [77,112,114], FIR could encumber TFIIH release, enable Pol II pausing and delay the subsequent progression to elongation. Indeed, FIR is an essential *MYC* repressor, as FIR knockdown is associated with *MYC* dysregulation ex vivo [143] and loss-of-function FIR mutations are associated with colorectal cancer displaying increased MYC abundance [144]. 

## 9. Dynamics of FUBP1, FIR and TFIIH Activity on the *MYC* Promoter

Serum-stimulation of mammalian tissue culture cells results in rapid activation of the endogenous *MYC* promoter and a pulse (i.e., up- followed by down-regulation) of *MYC* transcription [75]. Elegant ex vivo ChIP time-course experiments revealed the sequence of events on the *MYC* promoter following signal-stimulated *MYC* transcription, achieved by serum stimulation of previously starved human fibroblasts [143]. *MYC* enhancers and activators are first to load, enrichment for FUBP1 is subsequently detected, prior to decreased Pol II loading (i.e., consistent with release of paused Pol II) and increased abundance of *MYC* mRNA [143]. Following the peak in *MYC* mRNA levels, FUBP1 and FIR initially co-localise; however, once *MYC* returns to basal levels FUBP1 exits and only FIR is detected on FUSE. Thus, maximal activation of *MYC* transcription correlates with dissociation of RNA Pol II and recruitment of FUBP1 [143]. Pol II depletion from the transcription start site (TSS) and maximal enrichment for FUBP1, prior to the peak in *MYC* mRNA levels, is consistent with FUBP1 promoting RNA Pol II release to hyperactivate *MYC* transcription. 

The time course ChIP data supported findings from electrophoretic mobility shift assays (EMSA), demonstrating that FUBP1 and FIR binding occurs simultaneously; FIR addition enhances FUBP1 binding with FUSE; i.e., FUBP1’s affinity within the ternary FUBP1-FIR-FUSE complex is stronger than the FUBP1-FUSE interaction alone [133]. Moreover, the observation that FIR and FUBP1 enrichment is detected both on FUSE and proximal to the TSS suggests the FUBP1-FIR-XPB/TFIIH complex could generate a protein bridge between FUSE and TFIIH/Pol II bound to the TSS. Such a bridge would result in a closed topological DNA domain, to generate stress on the DNA duplex in the event of Pol II movement, which when transmitted as writhe would further melt FUSE to optimise the signle-stranded DNA (ssDNA) structure recognised by FIR. As FUBP1 leaves the complex, torsional strain would be lost and, as FIR engages, transcription would be returned to basal levels. The interaction between the FUBP1 activator and the repressor protein FIR can thus modify DNA topology and fine-tune *MYC* expression in response to signaling inputs.

## 10. Defective FIR-Dependent Repression of *MYC* in XPB-Related Disease

We predict that FUBP1 normally increases *MYC* transcription by stimulating XPB helicase activity and decreasing the frequency of Pol II pausing normally associated with the *MYC* promoter. FIR interacts with XPB/TFIIH to delay escape of Pol II from the promoter post-initiation. Such fine-tuning of transcription is essential for genes receiving multiple activation signals and requiring exquisite regulation like *MYC*, where recruitment alone would not provide sufficiently stringent control. Indeed, the early analysis of *MYC* dysregulation in Burkitt’s lymphoma revealed the importance of transcriptional regulation post-initiation, with precocious Pol II escape associated with *MYC*-driven pathology [77,112,114]. 

In addition to the essential function of TFIIH in basal transcription, the complex is essential for DNA excision repair [145]. Mutations in TFIIH subunits, including the XPB helicase, are associated with the inherited genetic disease Xeroderma Pigmentosum (XP), where failure of DNA repair and accelerated DNA damage drive carcinogenesis, especially in the skin to cause melanomas, squamous cell carcinomas, and basal cell carcinomas [145,146]. Although XP patient cells are responsive to other *MYC* transcriptional activators, they are defective in both FUBP1 activation and FIR repression [147]. In addition to chromosomal instability induced by defective DNA repair, the mutation at the 3′ end of XPB coding sequence in XP patients negates the XPB-FIR interaction and repression by FIR [147]. The FUBP1 and FIR enrichment normally detected on FUSE are no longer observed at the *MYC* TSS in cell lines derived from XP patients with C-terminally truncated XPB, suggesting a failure to form the FUBP1-FIR-XPB/TFIIH bridge as a consequence of the defective interaction between XPB and FIR. Interestingly, three different XP patients with mutations causing loss of the wild type C-terminus of XPB all display UV sensitivity, developmental and aging disorders, but have very different cancer predispositions (one severe, one moderate, one with no cancer) [148]. Together with the knowledge that all patients in these families have reduced NER, these observations suggest that cancer phenotypes are not due to NER defects per se, but likely due to a second mutation. Specifically, the cancer phenotypes could arise when XPB-dependent DNA repair defects alter hyperproliferative input(s); e.g., defective function of the FUBP1/FIR/TFIIH nexus would be predicted to disrupt repression of the *MYC* oncogene and contribute to XP-related neoplasia.

## 11. Defective *MYC* Repression and Tissue Overgrowth in *Drosophila* Models of XPB-Related Disease

**Figure 1:**
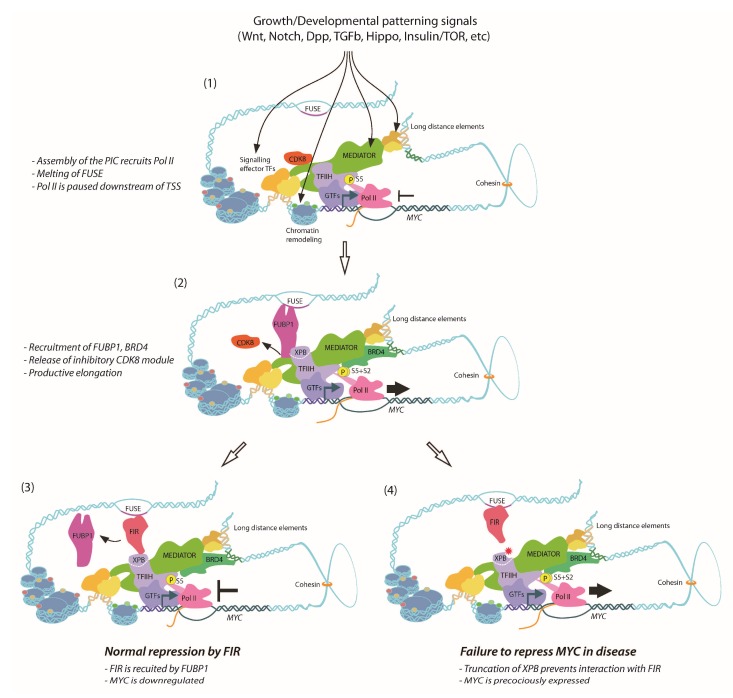
(**1**) For expression of *MYC* at basal levels, the pre-initiation complex (PIC) is assembled, consisting of the Mediator complex and general transcription factors (GTFs) which together recruit hypophosphorylated Pol II holoenzyme to be paused at the promoter. Recruitment of TFIIH promotes phosphorylation of Pol II at S5 residues. Upon activation by growth signals, chromatin is further remodeled, and downstream effector transcription factors (TFs) bind to enhancer elements, interacting with the *MYC* promoter via the Mediator complex, resulting in transcription and torsional strain on promoter, which promotes melting of the far upstream sequence element (FUSE) element. (**2**) In response to the growth signals, FUSE binding protein (FUBP1) recognizes and binds single-stranded FUSE, interacting with xeroderma pigmentosum type B (XBP) helicase subunit of TFIIH complex at the promoter and modulating nucleic acid architecture to facilitate exit of inhibitory cyclin-dependent kinase 8 (CDK8) module. Concurrently, bromodomain containing 4 (BRD4) interacts with the *MYC* promoter via Mediator complex, together with TFIIH promoting phosphorylation of Pol II at S2 residues. Thus, Pol II proceeds into productive elongation, and maximal expression of *MYC* is achieved. (**3**) FUBP1 recruits the FUBP-interacting repressor (FIR), which also binds to regulatory FUSE. FIR represses expression of *MYC* by negatively regulating TFIIH activity, reducing the rate of promoter escape by Pol II. **(4)** When the C-terminus of XPB is truncated due to mutations, interaction with FIR no longer occurs. Therefore, *MYC* promoter remains in a perpetual hyperactive state, increasing risk of tumourigenesis. TSS, transcription start site.

The *Drosophila* ortholog of FIR, Half pint (Hfp), behaves as a tumour suppressor, with reduced abundance of Hfp resulting in tissue overproliferation and larval overgrowth [149,150]. Haywire (Hay) is the *Drosophila* XPB homolog [151,152], and loss-of-function *Hay* mutants display phenotypes consistent with DNA repair and transcription defects [152,153]. Furthermore, Hay forms a complex with Hfp in vivo and co-ablation of *Hay* in *Hfp* loss-of-function cells reduces *dMYC* expression and cell growth [150], suggesting conservation between mammalian FIR-XPB and *Drosophila* Hfp-Hay in terms of transcriptional regulation of *MYC*. The *Drosophila* models therefore provide the means to dissect molecular mechanism(s) of defective repression of *dMYC* and tissue overgrowth associated with *Hfp* loss-of-function. Not only did *Hay/XPB* mutants—C-terminally truncated like the human disease alleles—strongly enhance cellular overproliferation and tissue overgrowth normally associated with the *Hfp* depletion, but also further impaired Pol II pausing and exacerbated *dMYC* derepression [154]. Thus, consistent with Pol II pausing attenuating *MYC* transcription, Hfp interacts with XPB/TFIIH to maintain a pool of engaged Pol II on the *dMYC* promoter in vivo [154]. An overview of the current understanding of *MYC* control by FUBP1/FIR is shown in Figure 1. Given the conserved nature of the FIR-XPB interaction, we would predict impaired Pol II pausing and defective transcriptional repression of *MYC* might contribute to hyperproliferation and cancer associated with XPB-related human diseases.

## 12. *Drosophila* FUBP1/Psi Interacts with Mediator to Control *MYC* Transcription

The capacity of the *MYC* promoter to integrate extracellular and developmental signals is fundamental to patterning of growth in multicellular animals. Although connections between signaling and patterning of *dMYC* transcription have been well delineated in *Drosophila* [44,79,155], until recently it was unknown whether signaling was integrated with *dMYC* transcription via P-element somatic inhibitor (Psi), the sole ortholog of the three mammalian FUBP proteins. Psi was originally ascribed the function of modulating splicing of transposable P-elements in *Drosophila* [156]. However, given that P-elements are a recent addition to *Drosophila melanogaster*, only entering the genome within the past 50 years [157], this function cannot reflect evolutionary pressures. Indeed, subsequent studies revealed broader roles for Psi in pre-mRNA splicing for many genes, including those required for courtship behaviour [158]. In accordance with Psi behaving in a functionally analogous manner to FUBP1, Psi is also essential for dMYC-dependent control of cell and tissue growth during *Drosophila* development [159]. The Psi interactome (determined by co-IP-mass spectrometry [160]) was predominantly comprised of Pol II transcriptional machinery [159]. Psi also has potent transcriptional activator capacity in vitro, mediated by conserved tyrosine-rich domains (YM1 and YM2 repeat motifs) in the C terminus; i.e., as observed for FUBP1 [161].

Of the Psi-interactors with designated functions in Pol II transcription (63% of the top 65 Psi-interactors), 32% were part of the chromatin-remodeling machinery, 12% gene specific transcriptional regulators, but by far most proteins (56%) comprised subunits of the transcriptional Mediator (MED) complex [159]. The MED complex interacts with the Pol II machinery to modulate transcription in all eukaryotes [162,163]. Although the MED complex is required for most (if not all) Pol II dependent transcription, the MED/CDK8 module can act as a sensor of developmental and environmental cues to activate specific transcriptional programs [164,165,166]. The MED complex bridges transcriptional enhancers and the Pol II machinery, to integrate developmental and environmental cues into specific transcriptional outcomes [167,168,169,170]. In line with this, MED responds to specific signaling networks to control developmental patterning in *Drosophila* [169,171,172,173,174]. In flies, *kohtalo* and *skuld*, which encode *Drosophila* homologs of the MED12 and MED13 subunits of the kinase module, are essential for the transcription of Wg/Wnt and Notch pathway targets and, thus, required to establish compartment boundaries of the wing imaginal disc [172,175,176]. More recently, a specific reduction in the expression of genes involved in wing margin formation was observed for *MED26* null mutant wing disc clones [173]. At the level of promoters, direct interplay between gene/tissue-specific Hox transcription factors and MED19 is essential for regulating expression of both embryonic and larval imaginal disc patterning genes [174]. A genetic screen for factors affecting wing growth, in the background sensitized with a copy of a *Minute* locus mutation, revealed a requirement for MED15 in expression of selected Dpp target genes, but not EGFR or Wg target genes [175].

In general, the large (~30 subunit) MED complex can behave either as an activator or inhibitor of Pol II-dependent transcription. The “small” or core MED complex is required for activation of Pol II transcription. The “large” complex has been predominantly characterised as a transcriptional repressor and it contains an additional 4 proteins; the kinase module comprising the Cyclin dependent kinase complex (CDK8/CycC) and 2 additional MED subunits (MED12, and MED13) [166]. The impaired growth phenotype associated with Psi depletion is suppressed following either co-depletion of subunits from the transcriptionally repressive CDK8/CycC kinase module or overexpression of core MED subunits, suggesting that Psi/dMYC-dependent tissue growth depends on MED abundance and activity [159]. Moreover, the decreased cell and tissue growth associated with Psi depletion is suppressed by dMYC overexpression and enhanced by co-knockdown of *dMYC*. In line with an activating role in *dMYC* transcription, Psi is required for maintaining *dMYC* mRNA at endogenous levels, with the latter being significantly decreased following Psi knockdown. In accordance with *dMYC* depletion being due to diminished transcriptional activity, ChIP revealed that depletion of Psi decreases enrichment of initiating Pol II (S5 phosphorylated) and elongating Pol II (S2 phosphorylated) across the *dMYC* gene. The observation that Psi activates from a single DNA binding site within a minimal promoter [159] (as observed for FUBP1 [161]), compared with most G4-activators that require tandem sites, suggests rather than acting at the level of Pol II recruitment, Psi drives *dMYC* transcription downstream of pre-initiation complex assembly.

Psi interacts with core Pol II machinery to maintain cell and tissue growth, which is of great interest given the proficiency of the MYC to drive cell growth [6,42]. We predict that *dMYC* dysregulation is capable of such potent modification of the Psi knockdown phenotype due to MYC’s capacity to act as a transcriptional amplifier [23,24]. In the context of rapidly proliferating wing disc cells, the major program of dMYC-modulated transcription will include genes required for cell and tissue growth. Thus, in the wing Psi and MED integrate growth signals to maintain developmentally regulated *dMYC* transcription, cell and tissue growth. Further studies are required to determine whether human FUBP1 also interacts with MED to modulate expression of the mammalian *MYC* oncogene. As even subtle increases in *MYC* expression (>2 fold) can promote the cell and tissue overgrowth fundamental to cancer initiation and progression, these observations will have implications for human disease [1,12]. 

## 13. MYC Control at A Distance—3D Genomic Architecture in *MYC* Regulation

Our understanding of transcriptional regulation has been greatly extended by elucidation of the 3-dimensional structure of the mammalian genome [177,178,179]. In particular, the capacity of promoter-enhancer loops to shape local DNA structure, revealed by techniques such as 3C and Hi-C, has enabled mapping of physically adjacent chromosome segments and determination of how their arrangement in topologically associated domains (TADs) affects gene expression [180]. Boundaries of TADs are generally delineated by cohesin-CCCTC-binding factor (cohesin-CTCF) loops [181]. Genes located within a common TAD naturally make contact more frequently and are more often co-expressed than genes located in different TADs, which suggests chromosomes arrange to establish environments enriched for transcriptional regulators to enable enhancer sharing within local insulated neighbourhoods [182,183]. 

The *Drosophila* genome is similarly arranged into physical domains that form visible structures in polytene chromosomes of the salivary glands [177,179]. *Drosophila* cohesin maintains genome structure by interacting with genes bound by paused Pol II to regulate gene expression within TADs [184]. *Drosophila* CTCF also binds distinct sites to enable mitotic bookmarking (i.e., maintenance of active and inactive chromatin marks [185]), in proliferating cells [186]. Highlighting the versatility of enhancer-promoter interactions, activation from an enhancer can occur bi-directionally in the *Drosophila* genome [187]. In the context of enhancer sharing, complexes such as MED, which can bridge enhancers to the general transcriptional machinery, will be key to integrating 3D interactions to modulate Pol II activity. 

In regard to the *MYC* locus, early studies identified CTCF as a repressor of the chicken *MYC* gene [188]. Regulation of *MYC* transcription by cohesin is also conserved in zebrafish [189]. Studies of long distance regulation in the mouse implicated the 8q24 region of the genome in regulation of the mammalian *Myc* gene [190,191]. The recent efforts, focused on the role TADs play in chromosome organization, revealed that mutations in CTCF binding sites in nonmalignant cells can initiate expression of oncogenes, including *MYC,* located within the insulated neighbourhood associated with T-cell acute lymphoblastic leukemia (T-ALL) pathogenesis [192]. Moreover, looping of the *MYC* locus is rearranged as a consequence of infection with the lymphoma-associated Epstein-Barr virus (EBV) [193]. Transactivators encoded by the virus hijack endogenous transcription machinery by recruiting SWI/SNF remodelers, to enable *MYC* promoter interactions with several upstream long range enhancers (ranging from -556kb to -168kb upstream of TSS) to drive *MYC* activation [193]. Thus, by allowing distant enhancers to come in proximity with promoters from ubiquitously expressed genes, disruption of TADs associated with *MYC* transcriptional control could drive cancer initiation. There are emerging links between the 3D genomic architecture and developmental signaling inputs, with NOTCH-mediated *MYC* activation occurring through direct binding at long-distance elements capable of forming loops with the *MYC* promoter [94,194]. Future studies investigating the response of the genome in 3D to signaling inputs implicated in *MYC* patterning, both specifically on the *MYC* locus and more globally, are awaited with great interest.

## 14. Targeting MYC for Cancer Treatment

Links to upstream pathways that input *MYC* transcriptional regulators would be informative for many current cancer therapies, where inhibition of signaling pathway components (particularly kinases) results in rapid development of drug resistance [195,196]. Commonly, cancer cells become resistant by accumulating mutations that activate a parallel pathway, ultimately providing compensatory signals that converge on common downstream effectors [197,198]. Therefore, co-targeting MYC in addition to upstream oncogenic signaling pathways would provide an avenue for treatment. 

As an excellent proof of principle, MYC can be targeted in transgenic mouse models of disease by expression of the dominant negative Omomyc [199,200]. This basic helix-loop-helix (bHLH) protein, which is able to form heterodimers with endogenous MYC, lacks capacity to bind DNA and thus interferes with MYC-MAX association and transcriptional activation, but enables MYC to still interact with the repressor network via MIZ-1 [200]. Omomyc was efficacious at eliminating Ras-driven adenocarcinoma in mice [201,202], and more recently global expression of Omomyc has been used to successfully treat Ras-induced astrocytoma in mouse models [203]. Although expression of Omomyc did reduce proliferation in normal tissues, it was generally well tolerated, and thus provides evidence that widespread dampening of MYC activity could be an effective cancer treatment. However, other attempts to target MYC protein itself have proven to be challenging [204], and individually inhibiting targets of MYC is difficult due to their number and variety [205]. Thus, targeting regulatory networks that control *MYC* transcription could be a promising treatment avenue.

The potential success of targeting *MYC* at the transcriptional level has been demonstrated using the bromodomain and extraterminal (BET) protein inhibitor JQ1. Although initially explored as a means of modulating MYC activity by interfering with *MYC*-associated acetylated histones, JQ1 regulates BRD4 to limit *MYC* expression in cancer cells [206]. BRD4 modifies *MYC* transcription by binding a super-enhancer 1.7Mb downstream of the *MYC* promoter [207,208] (Figure 1), providing another mechanism for *MYC*-specific activation by a factor originally characterized as playing general transcriptional co-activator roles [209]. Moreover, treatment with JQ1 kills T-ALL leukaemia cells with acquired resistance to γ**-**secretase, an inhibitor of NOTCH-mediated *MYC* activation [210] which is known to operate through control of promoter-enhancer looping [94,194].

Through 4C mapping, the long-distance BRD4-bound enhancer was found to form a loop preferentially interacting with the *MYC* promoter [207], and BRD4 drives *MYC* transcription by associating with the MED complex and the transcriptional elongation factor p-TEFb [211,212,213]. However, inhibition of BRD4 by JQ1 abolishes binding of the entire MED complex at multiple sites across the genome of acute myeloid leukemia (AML) cells, including the *MYC* promoter [214]. Consequently, BRD4 inhibition or downregulation results in cell cycle exit and differentiation, which can be replicated by knockdown of certain MED subunits e.g., MED23 and MED13 [214]. Tissue-specific requirements for MED23 have also been reported in Ras-driven lung tumorigenesis [215]. Future studies investigating whether Ras-driven tumours in the lung are sensitive to *MYC* modulation via BRD4 treatment would therefore be of great interest. 

## 15. Concluding Remarks and Future Considerations

Here we have brought together a snapshot of the vast web of signals integrated by the *MYC* promoter, with a focus on mechanisms required to fine-tune promoter output. In particular, the interplay between the two non-canonical *MYC* transcriptional regulators FUBP1 and FIR provides a mechanism for integration of cellular signals by enabling both rapid Pol II release (via FUBP1) and reestablishment of pausing (via FIR). The importance of these single-stranded nucleic acid binding proteins to *MYC* control in vivo is highlighted by their conservation between mammals and invertebrates, with *Drosophila* FIR/Hfp being essential for repression of *dMYC* [149,150,154], and Psi/FUBP1 required to maintain *dMYC* mRNA levels under signaling conditions conducive to growth in the fly wing [159]. The capacity of Psi/FUBP1 to modulate cell and tissue growth through interaction with the MED complex would provide a hub for integration of the developmental signaling inputs essential for the patterning of *MYC* transcription. 

Intriguingly, the bromodomain complex proteins were also detected in complex with Psi/FUBP1 in *Drosophila* mass spectrometry screens [160], including the first identified bromodomain protein Brm and the core members of the Brm complex, Moira and Ebi [159]. Investigation of the physiological importance of this interaction is compelling, particularly whether Psi/FUBP1 plays a role in recruiting BRD4/Brm to the *MYC* promoter to elicit *MYC*-specific regulatory effects and/or modifies BRD4/Brm activity in order to promote release of Pol II. In addition, 4C analysis of the *Drosophila dMYC* promoter will undoubtedly identify long-distance elements interacting with the Brm complex, MED and Psi/FUBP1. 

Recently the RNA-recognition motif (RRM)-containing protein B52, orthologous to mammalian Serine/arginine-rich splicing factors (SRSF1-6), was implicated in regulating *dMYC* expression and promoting cell growth [216]. B52 is essential for viability in *Drosophila* development [217], B52 overexpression increased *dMYC* promoter activity measured by *dMYC-lacZ* reporter activity and enrichment of phosphorylated Pol II across the *dMYC* promoter [216]. Determining whether B52, like the KH domain protein FUBP1 and RRM protein FIR, also possesses capacity to sequence-specifically bind ssDNA at the *MYC* promoter to modulate DNA topology and Pol II activity will be extremely important. Of great interest will be investigation of potential interactions with MED and the other single-stranded DNA-binding proteins implicated in *MYC* transcription, i.e., FUBP1/Psi and Hfp/FIR. We therefore invite future studies interrogating mechanisms by which FUBP1/Psi, FIR/Hfp and B52 sense the cellular signaling environment and how these factors modulate *MYC* promoter architecture to control cell and tissue growth during development, as these will shed light on MYC-dysregulation in cancer.
